# Low-density Lipoprotein Cholesterol Reduction Therapies for Secondary Prevention in Patients with Stroke: A Network Meta-analysis

**DOI:** 10.2174/1570159X22666231020093035

**Published:** 2023-10-20

**Authors:** Xing Wang, Jun Zheng, Yuqi Chen, Chao You, Lu Ma

**Affiliations:** 1 West China Hospital, Sichuan University, Chengdu, Sichuan 610041, PR China;; 2 West China Brain Research Centre, Sichuan University, Chengdu, Sichuan 610041, PR China

**Keywords:** Ischemic stroke, hemorrhagic stroke, low-density lipoprotein, PCSK9, secondary prevention, cardiovascular events

## Abstract

**Background:**

Patients with previous strokes are at a higher risk of stroke recurrence. Current guidelines recommend a range of low-density lipoprotein cholesterol (LDL-C)-lowering treatments to reduce the risk of recurrent stroke. However, the optimal agent for decreasing LDL-C to lower the risk of recurrent stroke remains unclear. This study aimed to assess the relative effects of various LDL-C -lowering agents for secondary stroke prevention.

**Methods:**

Several databases were searched from inception up to 2022. Only randomized controlled trials that compared different LDL-C-lowering agents in adult patients with previous strokes were included. The primary endpoint was a recurrent stroke. The surface under the cumulative ranking curve (SUCRA) was also applied to estimate the overall ranking probability of the treatment agents for each outcome.

**Results:**

Overall, nine trials comprising 17,226 patients were included. Ezetimibe plus statins (RR: 0.56, 95% CrI: 0.35-0.87) and statins alone (RR: 0.90, 95% CrI: 0.81-1.00) reduced the risk of stroke recurrence. Ezetimibe plus statins was superior to statins alone in decreasing the incidence of recurrent stroke (RR: 0.62, 95% CrI: 0.39-0.95). However, treatment with statins was related to an increased risk of hemorrhagic stroke compared to placebo (RR: 1.57, 95% CrI: 1.13-2.21). All agents were related to a decreased incidence of major adverse cardiovascular events.

**Conclusion:**

Treatment with ezetimibe plus statins was suggested as the most efficacious in decreasing the incidence of recurrent stroke. The analysis also revealed that statin monotherapy was related to an increased risk of hemorrhagic stroke.

## 1 INTRODUCTION

Stroke is identified as the second highest cause of death in developed areas and is a significant contributor to permanent disability [1, 2]. The estimated number of strokes worldwide has doubled in the past two decades [1]. The average long-term incidence of stroke recurrence after an initial transient ischemic attack (TIA) or stroke is approximately 10% at one year and increases to 40% at ten years [3]. Moreover, patients with previous strokes are at a higher risk of subsequent cardiovascular diseases, such as myocardial infarction (MI), leading to an increased risk of death due to vascular causes [4, 5]. However, secondary stroke prevention is currently suboptimal, with only 34.4% of stroke patients achieving lipid control targets, according to the EUROASPIRE III survey [6]. Therefore, there is an urgent need to improve risk factor control and secondary prevention in patients suffering from stroke.

In addition to antithrombotic and antihypertensive therapy, lipid-lowering therapy is an essential component of stroke prevention. Because the mechanisms underlying atherosclerosis are usually consistent across different regions of the vasculature, lipid-lowering therapy can reduce the risk of ischemic stroke as well as coronary events [7-9]. Robust evidence from clinical trials suggests that a range of low-density lipoprotein cholesterol (LDL-C)-lowering treatments, such as proprotein convertase subtilisin/kexin type 9 (PCSK9) inhibitors, statins, and ezetimibe, has shown promising efficacy in decreasing the risk of ischemic stroke [10, 11]. Specifically, in combination with other preventive measures, a 1 mmol/L decrease in plasma LDL-C results in a 20% reduction in the incidence of stroke over five years [12].

Intensive LDL-C-modifying treatment is recommended by the 2019 European Society of Cardiology (ESC) and European Atherosclerosis Society (EAS) guidelines to decrease the incidence of stroke recurrence in patients with TIA or ischemic stroke [13]. However, some concerns exist due to unexpected adverse events. For example, statin therapy may lead to a slight increase in the risk of bleeding events, but the evidence concerning this risk is inconclusive [14-16]. Moreover, the optimal agent for LDL-C reduction to reduce the incidence of recurrent stroke remains undetermined.

In this study, we used the Bayesian network approach to assess the effects and safety of various LDL-C reduction agents used for secondary stroke prevention. The comparative effects and ranking probabilities of various treatment agents were estimated. Our findings would be helpful for clinicians to guide patient management and for the development of clinical guidelines.

## 2 METHODS

### 2.1 Guidance and Protocol

This work was established according to the Preferred Reporting Items for Systematic Reviews and Meta-analyses for Network Meta-Analyses Extension Statement [17]. This study was registered in the OSF portal (https://osf.io/xgpcy) and PROSPERO database (CRD42022333817).

### 2.2 Eligibility Criteria

The studies were screened for eligibility based on their study population (all or a subset of adult patients aged ≥ eighteen years with previous stroke or TIA), interventions, and comparisons (statins *vs.* placebo, PCSK9 inhibitors with statins *vs.* statins alone, ezetimibe with statins *vs.* statins alone), outcomes (primary endpoint of recurrent stroke, secondary endpoints of ischemic stroke, hemorrhagic stroke, major adverse cardiovascular events (MACEs), cardiovascular mortality, and myocardial infarction), follow-up duration (at least one year or 48 weeks), and study design (randomized clinical trials).

### 2.3 Search Strategy

A thorough literature search was conducted in Ovid MedLine, Ovid EMBASE, and Cochrane CENTRAL from inception to May 19, 2022. Table **S1** in the Supplemental materials details the search strategy. In addition, the references of the included trials and relevant reviews on similar topics were searched to identify additional studies. Furthermore, the WHO Clinical Trials Registry Platform and the US National Library of Medicine of Clinical Trials Registry Portal were searched to identify ongoing randomized controlled trials.

### 2.4 Study Selection and Data Extraction

Two authors independently screened the titles and abstracts of all publications after removing duplicates. The full-text versions of studies were screened when both authors agreed on the study’s eligibility. Then the study characteristics of the eligible trials were extracted. Discrepancies were addressed through discussion among the study group. In cases of unclear or missing information, the corresponding author of the relevant study was contacted to request further information.

### 2.5 Quality Assessment

The risk of bias in the included trials was assessed across seven domains using the Cochrane Collaboration Risk of Bias tool [18]. Briefly, each study's overall risk of bias was rated as unclear, low, or high. The quality of evidence for each outcome was judged using the Grading of Recommendations Assessment, Development, and Evaluation (GRADE) tool [19]. The overall quality of evidence for each outcome was rated as high, moderate, low, or very low.

### 2.6 Data Synthesis

The present analysis was conducted using a consistency model to incorporate direct and indirect comparisons, and the method was described elsewhere [20]. Briefly, the models were developed based on 40,000 iterations after 15,000 iterations of grinding. We used the ranking functions to rank the intervention level of various treatment agents for each outcome, and the ranking results are presented with the surface under the cumulative ranking curve (SUCRA) values. We analyzed dichotomous variables as risk ratios (RRs) with related 95% credible intervals (CrIs) and continuous variables as the mean differences (MDs) with 95% CrIs. Heterogeneity in the model was assessed using the *I^2^* test. We assessed publication bias using the Harbord regression test if more than ten trials were synthesized [21].

The present analyses were completed using the relevant packages in R software (version 4.2.1) and Review Manager (version 5.4.0; Cochrane Collaboration). All *P* values were two-sided, and a value smaller than 0.05 was statistically significant. Analyses for all outcomes were performed for the intention-to-treat (ITT) population.

## 3 RESULTS

### 3.1 Characteristics of the Included Studies

The systematic electronic literature search yielded 1,253 publications (Fig. **S1**). After excluding studies according to the prespecified criteria, nine trials, including 17,226 patients, were included in the analyses [22-30].

The details of the eligible randomized controlled trials are shown in Table **1**. These trials were published from 1999 to 2020. The number of participants in each trial ranged from 122 to 5,337. The age of the patients ranged from 59 to 68 years. The proportion of females ranged from 25% to 56%. Two trials compared PCSK9 inhibitors with statins to statins alone, one compared ezetimibe with statins to statins alone, and six trials compared statin monotherapy to placebo.

#### 3.2 Recurrent Stroke

**Fig. (2):**
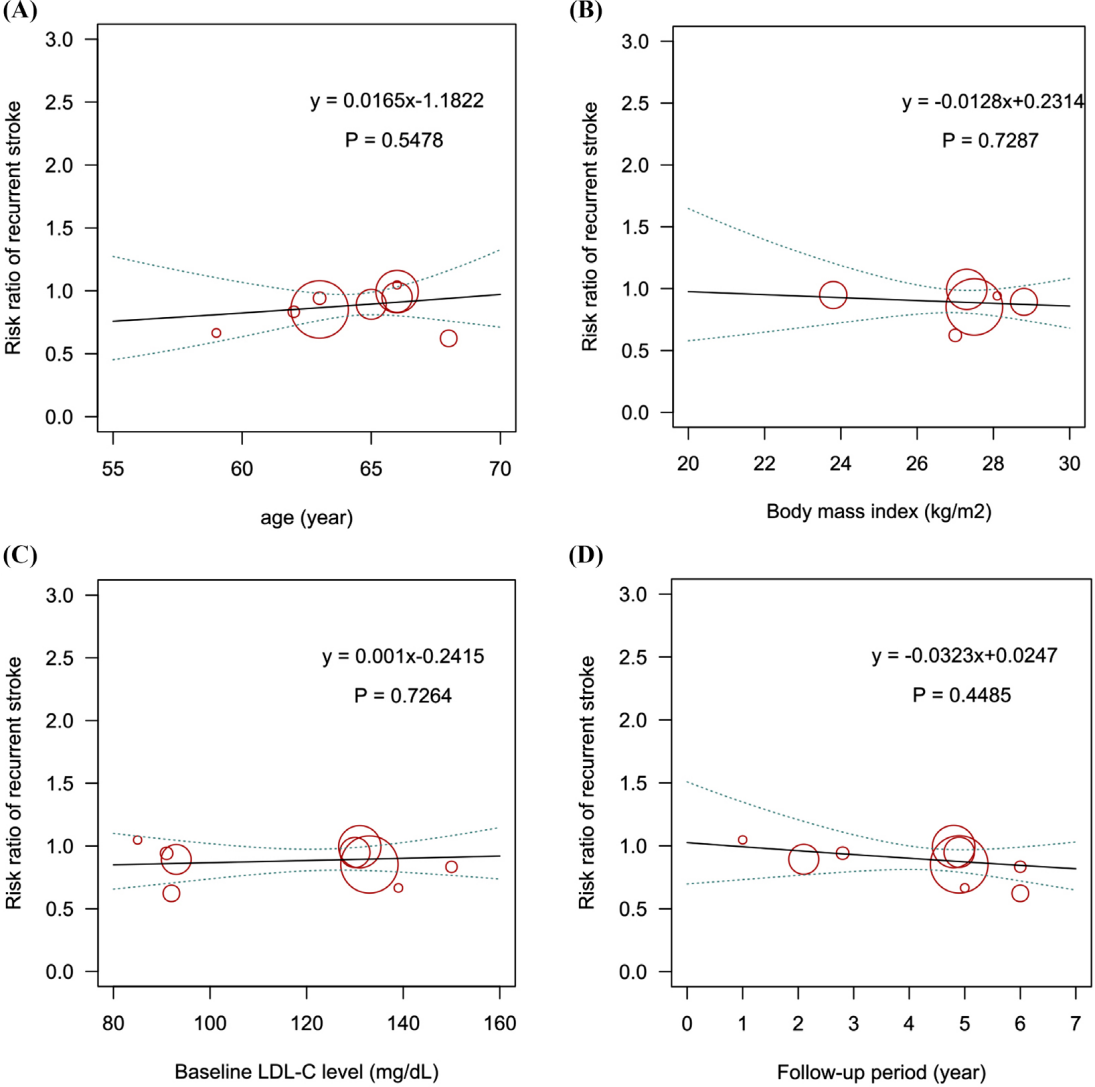
Meta-regression analysis for the interaction of (**A**) age, (**B**) BMI, (**C**) baseline LDL-C level, and (**D**) follow-up period on the risk of recurrent stroke. BMI: body mass index; LDL-C: low-density lipoprotein cholesterol.

Nine trials with 17,226 patients reported data on recurrent stroke (Figs. **S2-3**). The comparative effects of different LDL-C-lowering agents on stroke recurrence are shown in Fig. (**1**). Ezetimibe plus statins was identified as the best regimen for preventing stroke recurrence (RR: 0.56, 95% CrI: 0.35-0.87; SUCRA: 0.97) compared to placebo, followed by PCSK 9 inhibitors plus statins (RR: 0.82, 95% CrI: 0.63-1.06; SUCRA: 0.60) and statins alone (RR: 0.90, 95% CrI: 0.81-1.00; SUCRA: 0.40). Ezetimibe with statins was preferable to statins alone in decreasing the risk of stroke recurrence (RR: 0.62, 95% CrI: 0.39-0.95). Next, meta-regression analyses were conducted to assess the impacts of the follow-up period, body mass index (BMI), age, and baseline LDL-C level on the risk of stroke recurrence. The results showed an association between increased risk and older age, although this trend did not achieve statistical significance (*P*: 0.55, Fig. **2**).

Subgroup analyses were conducted for patients with ischemic stroke (Table **2**). Overall, five trials with 12,511 patients were pooled. The results showed that ezetimibe plus statins (RR: 0.56; 95% CrI: 0.35-0.87; SUCRA: 0.97) was associated with a decreased risk of stroke recurrence. This regimen also ranked first in reducing the incidence of stroke recurrence, followed by PCSK9 inhibitors plus statins (SUCRA: 0.60) and statins alone (SUCRA: 0.40).

#### 3.3 Ischemic Stroke

Six trials with 15,791 patients reported data on ischemic stroke (Fig. **S4**). The comparative effects of different LDL-C-lowering agents on ischemic stroke are shown in Fig. (**3A**, **3B**). Ezetimibe plus statins was identified as the best regimen for preventing ischemic stroke compared to placebo (RR: 0.44, 95% CrI: 0.26-0.72; SUCRA: 0.99), followed by PCSK9 inhibitors plus statins (RR: 0.76, 95% CrI: 0.55-1.05; SUCRA: 0.56) and statins alone (RR: 0.83, 95% CrI: 0.73-0.94; SUCRA: 0.43). Ezetimibe plus statins was superior to statins alone in reducing the incidence of ischemic stroke (RR: 0.53, 95% CrI: 0.32-0.86).

#### 3.4 Hemorrhagic Stroke

**Fig. (4):**
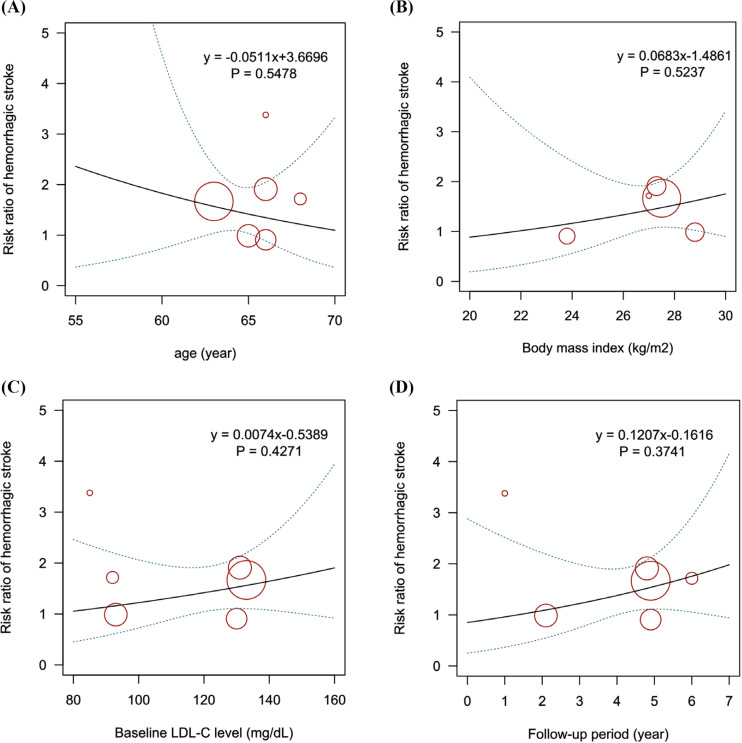
Meta-regression analysis for the interaction of (**A**) age, (**B**) BMI, (**C**) baseline LDL-C level, and (**D**) follow-up period on the risk of hemorrhagic stroke. BMI: body mass index; LDL-C: low-density lipoprotein cholesterol.

Six trials with 15,791 patients reported data on hemorrhagic stroke (Fig. **S5**). The comparative effects of different LDL-C-lowering agents on hemorrhagic stroke are shown in Fig. (**3C**, **3D**). Ezetimibe plus statins was the most likely regimen to cause hemorrhagic stroke compared to placebo (RR: 2.87, 95% CrI: 0.64-15.33; SUCRA: 0.82), followed by statins alone (RR: 1.57, 95% CrI: 1.13-2.21; SUCRA: 0.57) and PCSK9 inhibitors plus statins (RR: 1.56, 95% CrI: 0.69-3.56; SUCRA: 0.53). Meta-regression analyses were conducted to evaluate the impacts of age, BMI, baseline LDL-C level, and follow-up period on the incidence of hemorrhagic stroke. There was a trend toward a reduced risk of hemorrhagic stroke with increasing age. The results also suggested that patients with higher BMI had a higher risk of hemorrhagic stroke, although the trend did not achieve statistical significance (Fig. **4**).

#### 3.5 Risk of Bias and Quality of Evidence

Figs. (**S6** and **7**) present the key findings of the risk-of-bias assessments. Four trials were identified to have an overall low risk of bias. The other trials were identified to have an overall high risk of bias. The key findings of the GRADE evaluation of certainty for stroke outcomes are presented in Table **S2** (Supplemental material). In general, the quality of evidence for the primary outcome was judged to be moderate to high.

#### 3.6 Other Outcomes

**Fig. (5):**
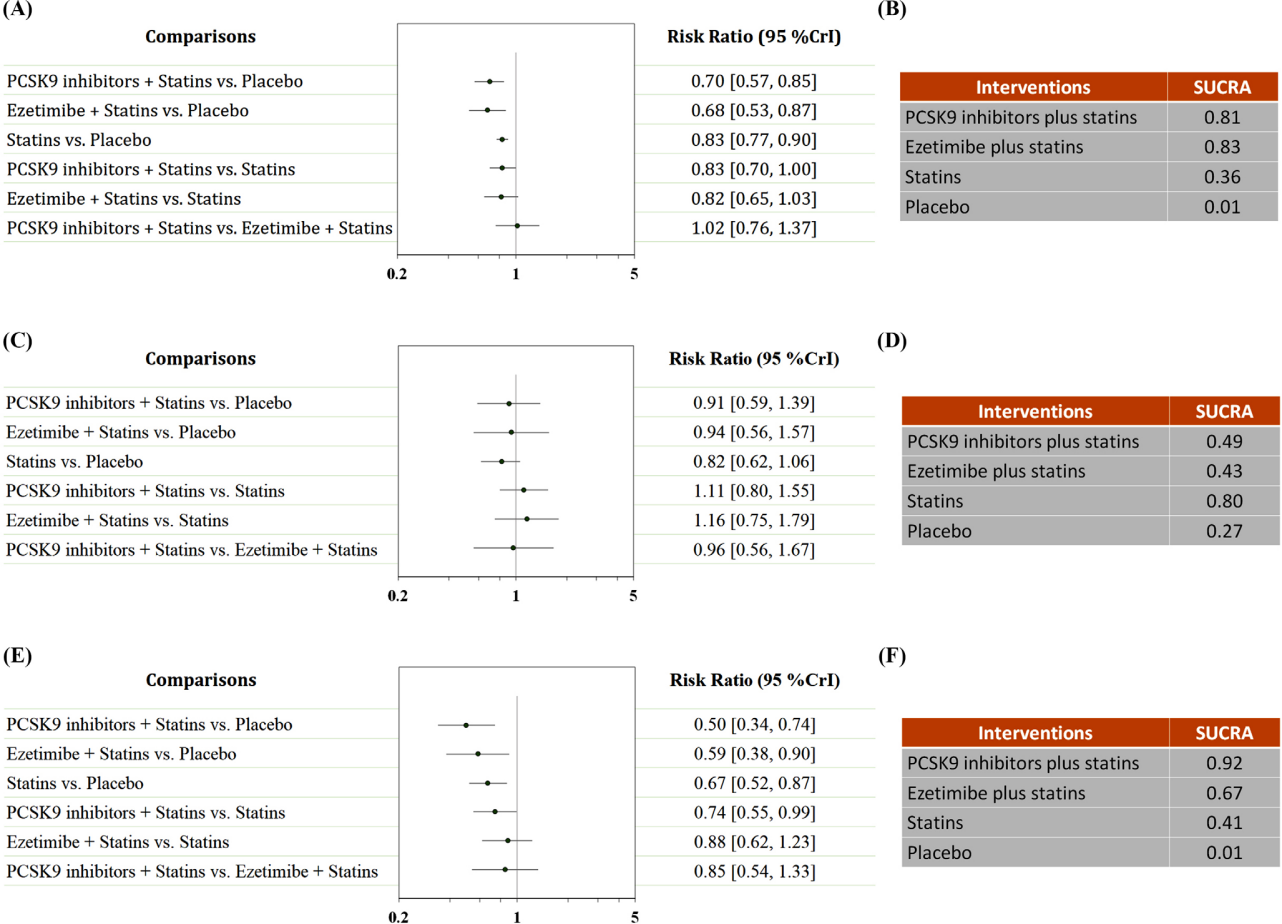
Network analysis for other outcomes. (**A**) The forest plot for MACE. (**B**) The SUCRA value of each treatment for MACE. (**C**) The forest plot for cardiovascular mortality. (**D**) The SUCRA value of each treatment for cardiovascular mortality. (**E**) The forest plot for myocardial infarction. (**F**) The SUCRA value of each treatment for myocardial infarction. MACE: major adverse cardiovascular event. CrI: credible interval. SUCRA: surface under the cumulative ranking curve.

Data on the other outcomes are presented in Fig. (**5**). All agents were associated with a decreased risk of MACEs (Fig. **5A**, **5B**). Ezetimibe plus statins was ranked first for this outcome (RR: 2.87, 95% CrI: 0.64-15.33 compared to placebo; SUCRA: 0.83), followed by PCSK9 inhibitors plus statins (RR: 0.70, 95% CrI: 0.57-0.85; SUCRA: 0.81) and statins alone (RR: 0.83, 95% CrI: 0.77-0.90; SUCRA: 0.36). We did not detect a significant difference between different treatment regimens in the reduction of the risk of cardiovascular death (Fig. **5C**, **5D**). All agents were associated with a decreased incidence of MI (Fig. **5E**, **5F**). PCSK9 inhibitors plus statins ranked first for this outcome (RR: 0.50, 95% CrI: 0.34-0.74 compared to placebo; SUCRA: 0.92), followed by ezetimibe plus statins (RR: 0.59, 95% CrI: 0.38-0.90; SUCRA: 0.67) and statins alone (RR: 0.67, 95% CrI: 0.52-0.87; SUCRA: 0.41). PCSK9 inhibitors plus statins were superior to statins alone in reducing the incidence of MI (RR: 0.74, 95% CrI: 0.55-0.99).

## 4 DISCUSSION

### 4.1 Summary of Findings

In the present study of nine trials with 17,226 participants, we compared the direct and indirect effects of medications on outcomes to provide updated evidence aimed at exploring the optimal agents for preventing stroke recurrence in people with previous strokes. In the present study, several important observations were made. First, ezetimibe plus statins ranked first in reducing the risk of recurrent stroke. Second, statins might increase the risk of hemorrhagic stroke. Third, all three treatment regimens were superior to placebo in decreasing the incidence of MACEs. Furthermore, meta-regression analysis suggested a trend toward a higher incidence of hemorrhagic stroke in patients with higher BMI.

Our study revealed that statins might increase the risk of hemorrhagic stroke, which could be explained by the following reasons. On the one hand, lower LDL-C levels may be associated with an increased risk of cerebral hemorrhage. For example, epidemiological studies have shown an association between low LDL-C levels and intracranial hemorrhage [31, 32]. Some researchers have even recommended avoiding statins in patients with a history of ICH [33, 34]. Second, in addition to their lipid-lowering effects, statins also have antithrombotic activity, which enhances fibrinolysis and anticoagulation by inhibiting platelet aggregation [35, 36]. Third, it is also important to note that different classes of statins have different effects on ICH; for example, this study showed that lipophilic statins were likely to increase the risk of hemorrhagic stroke (Fig. **S8**). Similar findings have been reported in previous studies [37, 38].

### 4.2 Comparison with Other Studies

Several meta-analyses have assessed the efficacy of lipid-modifying therapies for the primary and secondary prevention of ischemic stroke. One review revealed that PCSK9 inhibitors plus statins and ezetimibe decreased the incidence of ischemic stroke without increasing the incidence of hemorrhagic stroke [39]. Another systematic review showed that statins increased the risk of hemorrhagic stroke (RR: 1.15, 95% CI: 1.00-1.32), but PCSK9 inhibitors did not increase the risk (RR: 0.93, 95% CI: 0.58-1.51) [40]. Only one network meta-analysis assessed different statin types for the secondary prevention of stroke [41]. Evidence from this study indicated that patients treated with statins had an elevated risk of hemorrhagic stroke (0.6%) compared to those treated with placebo/no statins. In particular, the administration of 80 mg atorvastatin per day or 40 mg simvastatin per day should prompt careful consideration of the potential risk of hemorrhagic stroke. Previous studies on this topic have yielded similar results [42, 43].

### 4.3 Study Strengths and Limitations

To date, this study is the most extensive systematic review and the only network meta-analysis to examine the efficacy and safety of different LDL-C-lowering therapies for the secondary prevention of stroke. The large sample size of the present study gave credibility to our results because of the improved precision of estimation regarding the treatment effects. It allowed informed suggestions for choosing optimal treatment agents. In addition, we conducted subgroup analysis for selected patients with ischemic stroke and ranked the quality of evidence for stroke outcomes according to the GRADE method. Moreover, meta-regression analyses were conducted to test the effects of different variables on the risk of stroke recurrence and hemorrhagic stroke, which was helpful for future research.

There are a few limitations of this study that need to be discussed. First, participants and care providers were aware of the treatment allocation in some trials, resulting in an increased likelihood of bias regarding the assessment of outcomes in the absence of blinding. Second, we focused on long-term outcomes and included studies with a follow-up duration of at least one year of follow-up. Because the follow-up period varied across trials, we performed a meta-regression analysis to explore whether the follow-up duration influenced the effects of LDL-C-lowering therapies but did not observe any such relationship (*P:* 0.449). Third, most of the included trials did not provide data on LDL-C reduction; therefore, we were unable to assess the comparative effects of different treatment regimens (Fig. **S9**).

### 4.4 Study Implications

The ACC/AHA and ESC/EAS guidelines recommend intensive lipid-lowering therapy in persons with previous TIA or ischemic stroke [13, 44, 45]. However, the optimal type of LDL-C lowering agent has not been specified. Based on the moderate quality of evidence, we suggest that ezetimibe with statins might be more effective than monotherapy with statins in preventing stroke recurrence. Our analyses also suggest that a regimen of PCSK9 inhibitors with statins is likely preferable to statins alone to reduce the incidences of MACEs and MI. However, it is suggested that treatment with statins alone is associated with an increased incidence of hemorrhagic stroke. Clinicians should comprehensively evaluate drug efficacy, safety, and cost-effectiveness when choosing a specific agent.

## CONCLUSION

This network meta-analysis indicated that ezetimibe plus statins might be the most effective regimen for decreasing the incidence of recurrent stroke in people with previous stroke. Although statins have shown a superior effect in decreasing the incidence of recurrent stroke compared to placebo, attention should be given to the potential increased risk of hemorrhagic stroke that they may cause. Regarding cardiovascular events, although all the agents decreased the risk of major adverse cardiovascular events and myocardial infarction, PCSK9 inhibitors were found to be the most effective treatment agent compared with either placebo or statins. These findings provide novel and essential evidence for clinicians to guide therapeutic strategies.

## Figures and Tables

**Fig. (1):**
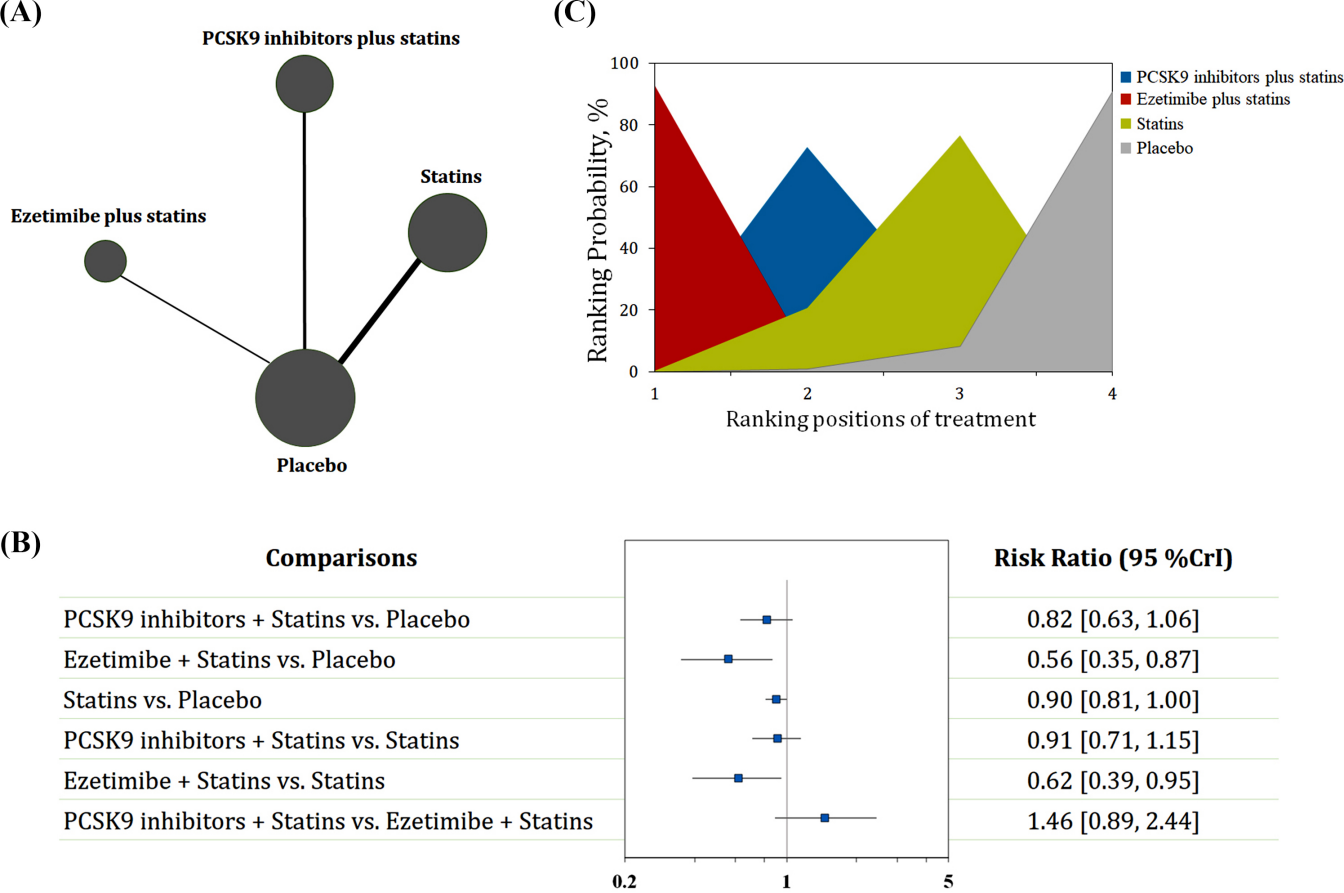
Summary of the recurrent stroke outcome. (**A**) Network plot of recurrent stroke. The width of the lines is proportional to the number of studies comparing every pair of treatments, and the size of each circle is proportional to the number of participants. (**B**) The forest plot shows the risk ratio (RR) and credible interval (CrI). (**C**) Ranking probability graph of each treatment agent. The SUCRA values for each treatment were as follows: 60% for PCSK9 inhibitor plus statins, 97% for ezetimibe plus statins, and 40% for statins alone. SUCRA: surface under the cumulative ranking curve.

**Fig. (3):**
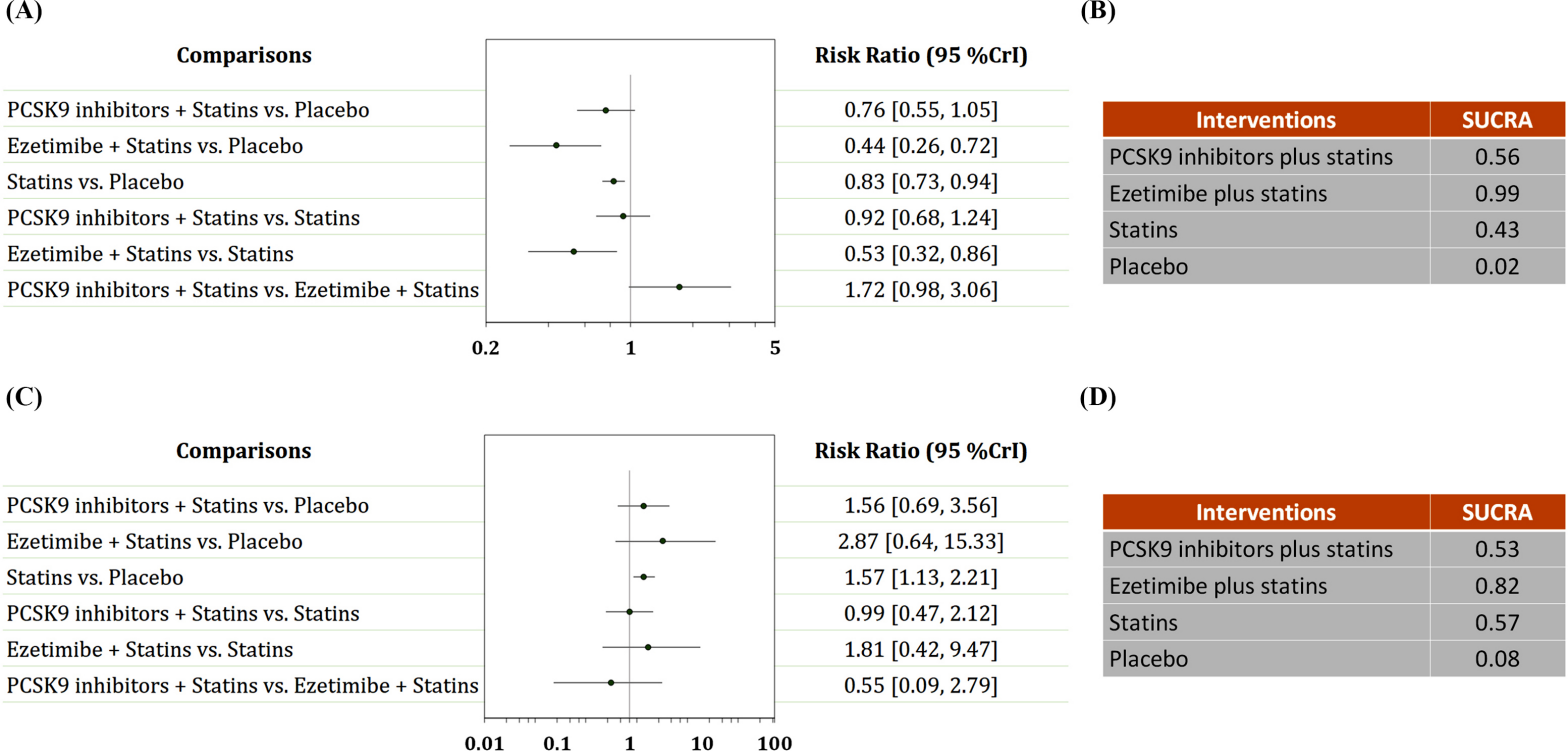
Network analysis for ischemic stroke and hemorrhagic stroke. (**A**) The forest plot for ischemic stroke. (**B**) The SUCRA value of each treatment for ischemic stroke. (**C**) The forest plot for hemorrhagic stroke. (**D**) The SUCRA value of each treatment for hemorrhagic stroke. CrI: credible interval. SUCRA: surface under the cumulative ranking curve.

**Table 1:** Characteristics of studies included in the systematic review.

**Trial**	**Patients, ** **n**	**% ** **Female**	**Age, ** **Years**	**BMI, ** **kg/m^2^**	**Baseline ** **LDL-C (mg/dl)**	**Follow-up Time**	**Comparisons**	**Characteristics of Patients**
Fourier 2020	5337	26	65	28.8	93	2.1 years	Evolocumab plus statins *vs*. statins	Subgroup of patients with history of ischemic stroke
Odyssey Outcomes 2019	944	32	63	28.1	91	2.8 years	Alirocumab plus statins *vs*. statins	Subgroup of patients with history of stroke
Improve-IT 2017	682	29	68	27	92	6 years	Ezetimibe plus simvastatin *vs*. simvastatin	Subgroup of patients with history of stroke
J-STARS 2015	1578	31	66	23.8	130	4.9 years	Pravastatin *vs*. placebo	Non-cardioembolic ischemic stroke
Yakusevich *et al*. 2012	183	56	66	NA	85	1 year	Simvastatin *vs*. placebo	First acute ischemic stroke
SPARCL 2006	4731	40	63	27.5	133	4.9 years	Atorvastatin *vs*. placebo	Non-cardioembolic stroke or TIA
HPS 2004	3280	25	66	27·3	131	4.8 years	Simvastatin *vs*. placebo	Subgroup of patients with history of cerebrovascular disease
LIPID 2000	369	NA	62	NA	150	6 years	Pravastatin *vs*. placebo	Subgroup of patients with history of stroke
CARE 1999	122	NA	59	NA	139	5.0 years	Pravastatin *vs*. placebo	Subgroup of patients with history of stroke

**Abbreviations:** NA: not available; BMI: body-mass index; LDL-C: low-density lipoprotein cholesterol; TIA: transient ischemic attack.

**Table 2:** Pooled RR and relative CrI derived from network meta-analysis with different treatment regimens in patients with ischemic stroke.

**Intervention**	**RR (95% CrI) Estimates Derived from NMA**						**SUCRA**		
	**PCSK9 Inhibitor Plus Statins *vs.* Placebo**	**Ezetimibe ** **Plus Statins *vs.* Placebo**	**Statins *vs.* Placebo**	**PCSK9 ** **Inhibitor Plus Statins *vs.* Statins**	**Ezetimibe Plus Statins *vs.* Statins**	**PCSK9 Inhibitor Plus Statins *vs.* Ezetimibe Plus Statins**	**PCSK9 Inhibitor Plus Statins**	**Ezetimibe Plus Statins**	**Statins**
Recurrent stroke	0.82 (0.63, 1.06)	**0.56 ** **(0.35, 0.87)**	**0.90 ** **(0.81, 1.00)**	0.91 (0.71, 1.15)	**0.62 ** **(0.39, 0.95)**	1.46 (0.89, 2.44)	0.60	0.97	0.40
Ischemic stroke	0.76 (0.55, 1.05)	**0.44 ** **(0.26, 0.72)**	**0.83 ** **(0.73, 0.94)**	0.92 (0.68, 1.24)	**0.53 ** **(0.32, 0.86)**	1.72 (0.98, 3.06)	0.56	0.99	0.43
Hemorrhagic stroke	1.56 (0.69, 3.56)	2.87 (0.64, 15.33)	**1.57 ** **(1.13, 2.21)**	0.99 (0.47, 2.12)	1.81 (0.42, 9.47)	0.55 (0.09, 2.79)	0.53	0.82	0.57
MACE	**0.70 ** **(0.57, 0.85)**	**0.68 ** **(0.53, 0.87)**	**0.83 ** **(0.77, 0.90)**	**0.83 ** **(0.70, 1.00)**	0.82 (0.65, 1.03)	1.02 (0.76, 1.37)	0.81	0.83	0.36
Cardiovascular mortality	0.91 (0.59, 1.39)	0.94 (0.56, 1.57)	0.82 (0.62, 1.06)	1.11 (0.80, 1.55)	1.16 (0.75, 1.79)	0.96 (0.56, 1.67)	0.49	0.43	0.80
MI	**0.50 ** **(0.34, 0.74)**	**0.59 ** **(0.38, 0.90)**	**0.67 ** **(0.52, 0.87)**	**0.74 ** **(0.55, 0.99)**	0.88 (0.62, 1.23)	0.85 (0.54, 1.33)	0.92	0.67	0.41

**Abbreviations:** RR: relative risk; CrI: credibility interval; SUCRA: surface under the cumulative ranking curve; NMA: network meta-analysis; PCSK9: proprotein convertase subtilisin/kexintype9; MI: myocardial infarction; MACE: major adverse cardiovascular event.

## LIST OF ABBREVIATIONS

LDL-C: Low-density Lipoprotein Cholesterol
RCTs: Randomized Clinical Trials
MACEs: Major Adverse Cardiovascular Events
RRs: Relative Risks
CrIs: Credible Intervals
TIA: Transient Ischemic Attack
PCSK9: Proprotein Convertase Subtilisin/Kexintype9
PRISMA: Preferred Reporting Items for Systematic Reviews and Meta-analyses
NMA: Network Meta-analyses
GRADE: Grading of Recommendations Assessment, Development, and Evaluation working group
MD: Mean Difference
SUCRA: Surface Under The Cumulative Ranking Curve
ITT: Intention-to-treat
BMI: Body Mass Index
MI: Myocardial Infarction

## STANDARDS OF REPORTING

PRISMA guidelines and methodology were followed.

## SUPPLEMENTARY MATERIAL

PRISMA checklist and supplementary material is available on the publisher’s website along with the published article.
