# Supplementary material for: Low-density Lipoprotein Cholesterol Reduction Therapies for Secondary Prevention in Patients with Stroke: A Network Meta-analysis
Source: Curr Neuropharmacol. 2023 Oct 20;22(12):2034–44. doi: 10.2174/1570159X22666231020093035 (PMC11333788; doi:10.2174/1570159X22666231020093035)
Supplement: Supplementary file 1 [file CN-22-2034_SD1.pdf]

Supplementary Material

Low-density Lipoprotein Cholesterol Reduction Therapies for Secondary Prevention in Patients with Stroke: A Network Meta-analysis

Xing Wang[1], Jun Zheng[1], Yuqi Chen[1], Chao You[1,2] and Lu Ma[1,*]

[1]*West China Hospital, Sichuan University, Chengdu, Sichuan 610041, PR China;* [2]*West China Brain Research Centre, Sichuan University, Chengdu, Sichuan 610041, PR China*

Table S1: Search Strategy.

OVID EMBASE (Adapted for Other Databases)	
1	exp Hydroxymethylglutaryl-CoA Reductase Inhibitors/ or exp Alirocumab/ or exp Evolocumab/ or exp Ezetimibe/
2	(statin* or PCSK9 inhibitor* or Ezetrol or Ezetimib). ab,kw,ti
3	#1 or #2
4	exp Stroke/ or exp Ischemic Attack, Transient/
5	(Cerebrovascular Accident* or Cerebral Stroke or Brain Vascular Accident* or Cerebrovascular Stroke* or Apoplexy or transient ischemic attack* or Transient Cerebral Ischemia*).ab,kw,ti
6	#4 or #5
7	#3 and #6
8	exp secondary prevention/
9	(Early Therap* or Secondary Prevention* or recurrent stroke* or stroke recurrence* or secondary stroke*). ab,kw,ti
10	#8 or #9
11	#7 and #10
12	exp randomized controlled trial/
13	(random* or blind* or placebo or trial*).ab,kw,ti.
14	#12 or #13
15	exp human/
16	#14 and #15
17	#11 and #16

1875-6190/24 $65.00+.00 **© 2024 Bentham Science Publishers**

Table S2. GRADE assessment of certainty for recurrent stroke.

	Quality of Evidence		
	**Recurrent Stroke**	**Ischemic Stroke**	**Hemorrhagic Stroke**
**Compared with Placebo**			
PCSK9 inhibitor plus statins	High	High	Moderate#
Ezetimibe plus statins	High	High	Low†
Statins	High	High	Moderate#
**Compared with statins**			
PCSK9 inhibitor plus statins	Moderate§	Moderate§	Low§#
Ezetimibe plus statins	Moderate§	Moderate§	Very low§†
**Compared with ezetimibe plus statins**			
PCSK9 inhibitor plus statins	Low§#	Very low§†	Very low§†

§: Rated down for serious indirectness

#: Rated down for serious imprecision

†: Rated down two levels for very serious imprecision

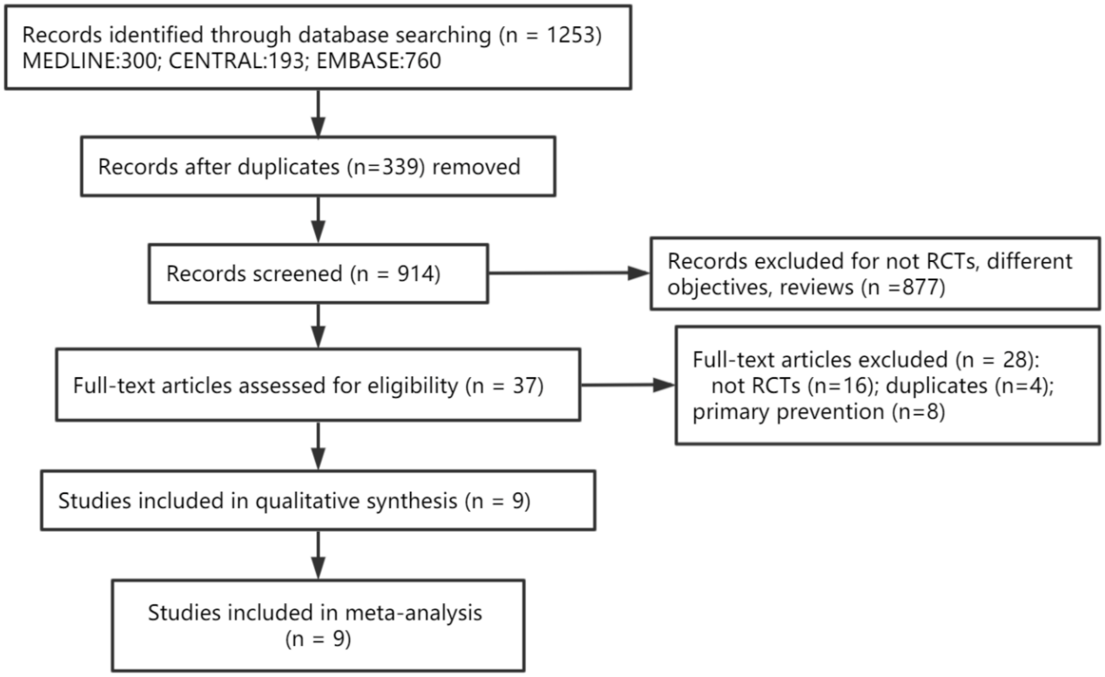

Figure S1: Study selection flowchart of randomized controlled trials.

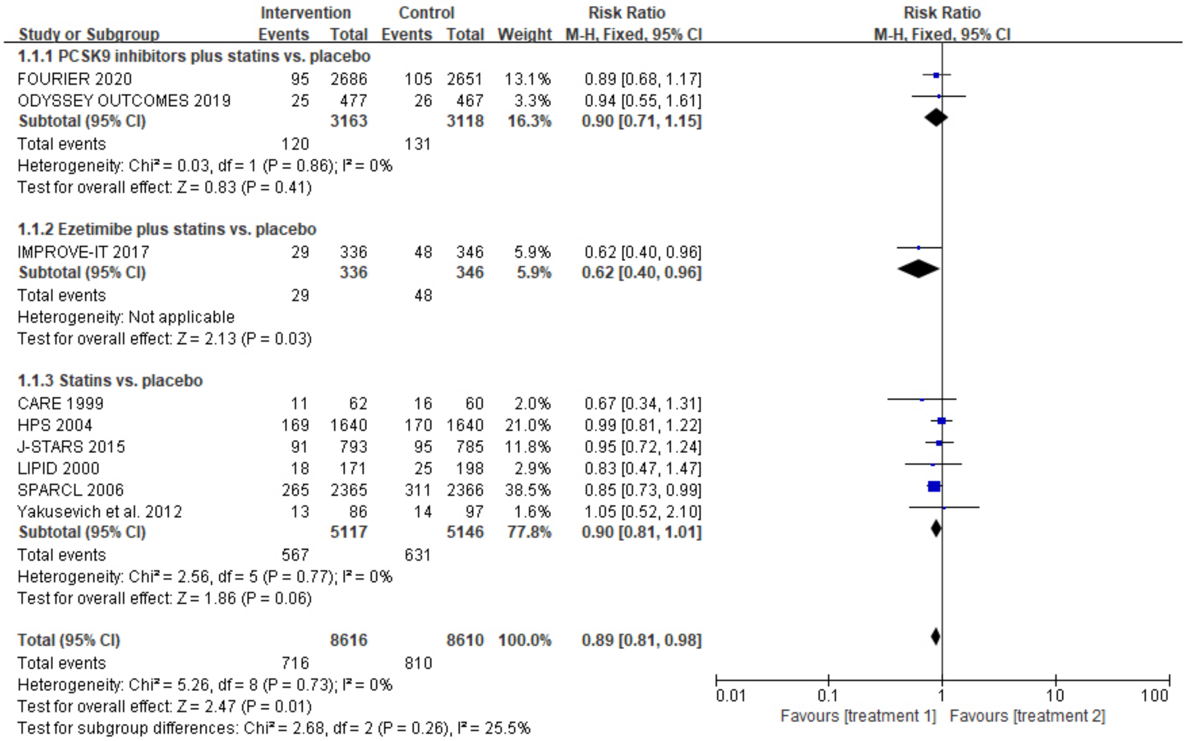

Figure S2: Forest plot of recurrent stroke in the direct meta-analysis.

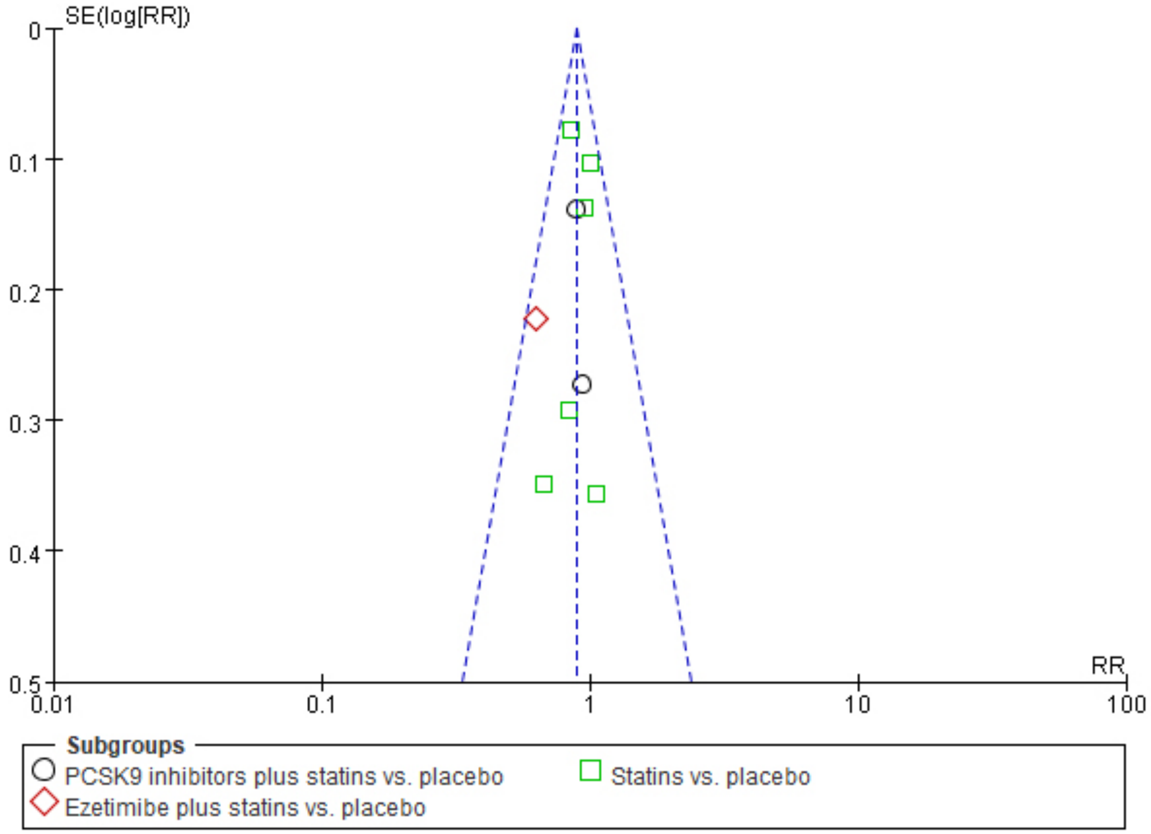

Figure S3: Funnel plot of recurrent stroke in the direct meta-analysis.

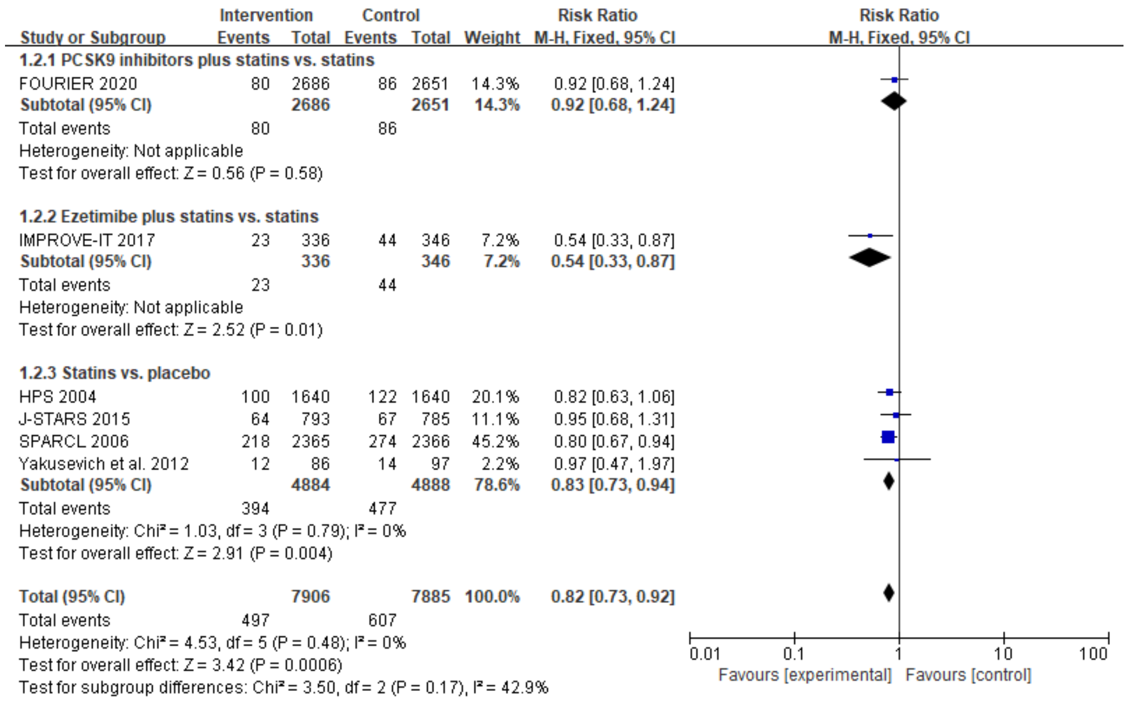

Figure S4: Forest plot of ischemic stroke in the direct meta-analysis.

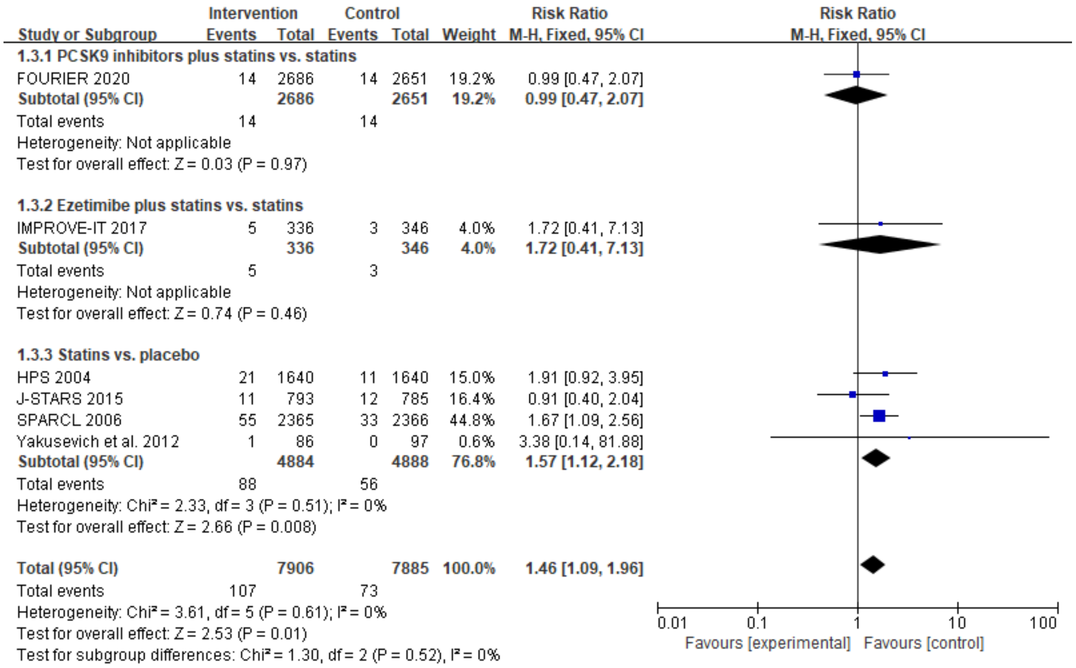

Figure S5: Forest plot of hemorrhagic stroke in the direct meta-analysis.

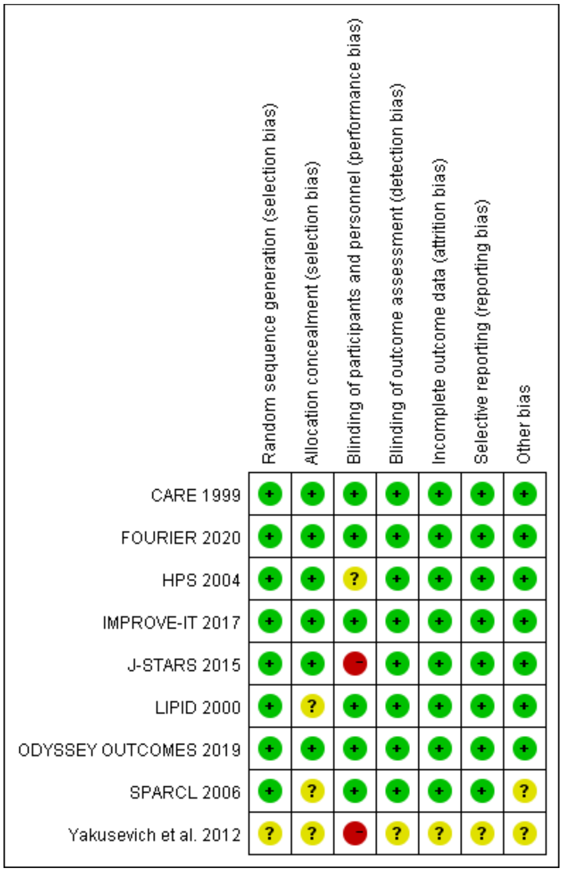

Figure S6: Risk of bias summary: review authors' judgements about each risk of bias item for each included study.

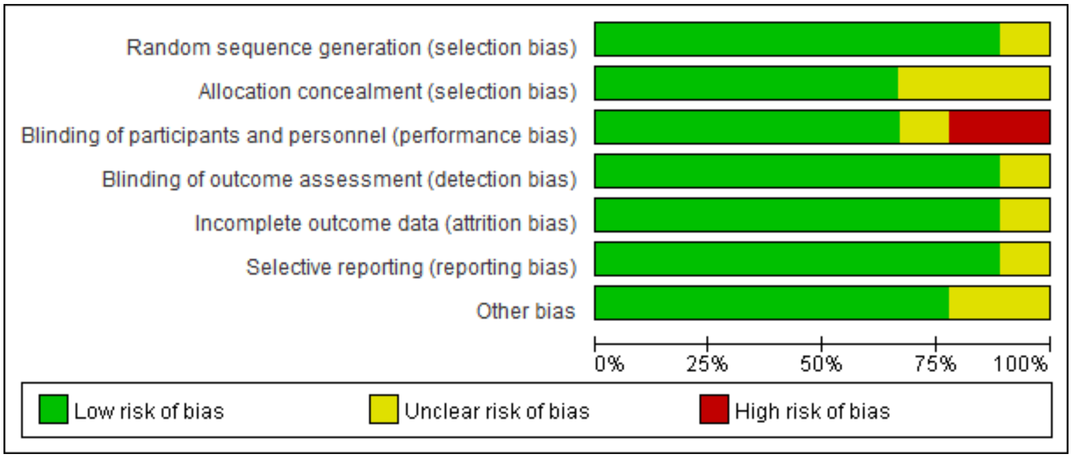

Figure S7: Risk of bias graph: review authors' judgements about each risk of bias item presented as percentages across all included studies.

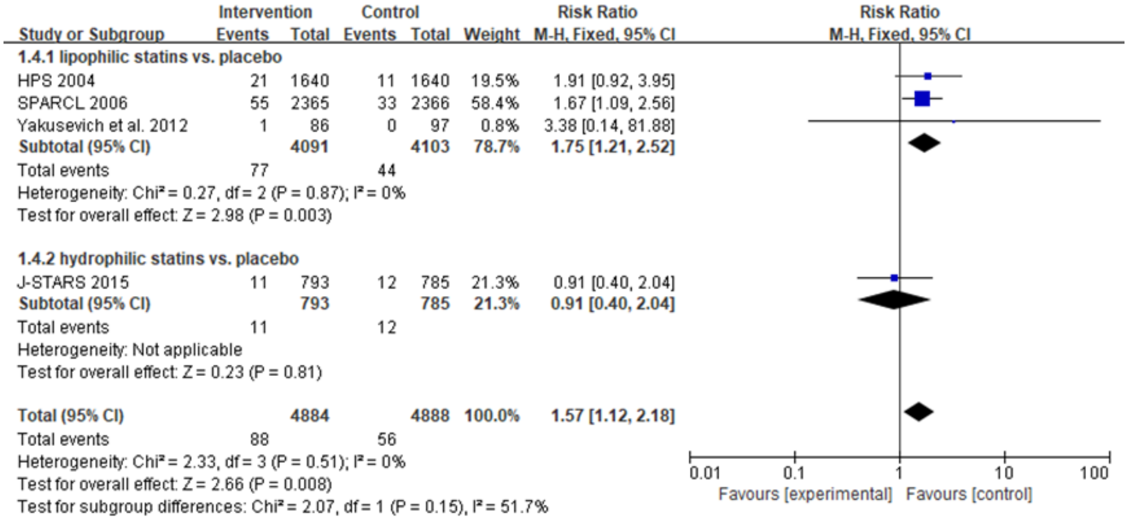

Figure S8: Forest plot of different classes of statins in the hemorrhagic stroke from the direct meta-analysis.

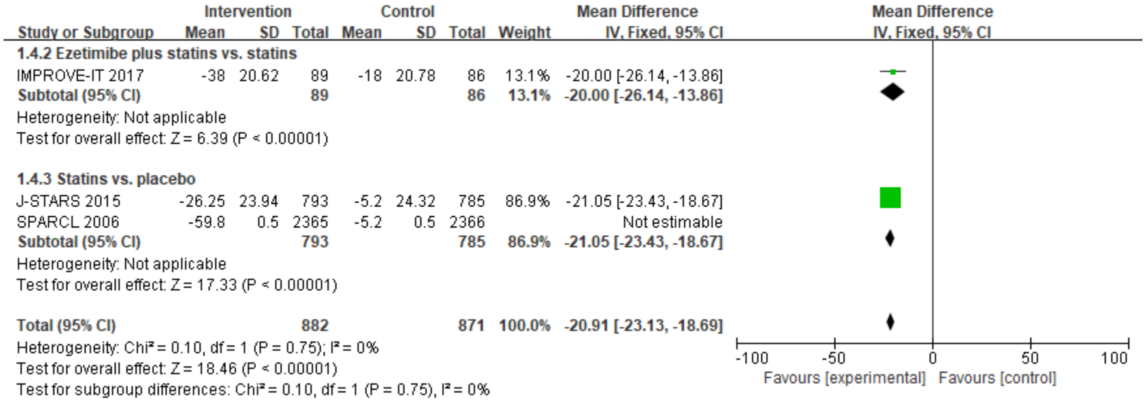

Figure S9: Forest plot of LDL-C reduction in the direct meta-analysis.
